# Evaluation of stem-like side population cells in a recurrent nasopharyngeal carcinoma cell line

**DOI:** 10.1186/s12935-014-0101-0

**Published:** 2014-10-09

**Authors:** Susan Ling Ling Hoe, Lu Ping Tan, Juliana Jamal, Suat Cheng Peh, Ching Ching Ng, Wen Cai Zhang, Munirah Ahmad, Alan Soo Beng Khoo

**Affiliations:** Molecular Pathology Unit, Cancer Research Centre, Institute for Medical Research, Jalan Pahang, 50588 Kuala Lumpur, Malaysia; Institute of Biological Sciences, Faculty of Science, University of Malaya, 50603 Kuala Lumpur, Malaysia; Faculty of Medical Sciences, UCSI University, 1 Jalan Menara Gading, UCSI Heights, 56000 Cheras, Malaysia; Genome Institute of Singapore, 60 Biopolis Street, #02-01, Genome, 138672 Singapore; Current address: Faculty of Medicine, University of Malaya, 50603 Kuala Lumpur, Malaysia

**Keywords:** Side population, Nasopharyngeal carcinoma, Slow-cycling, Stem-like, Cancer stem cells

## Abstract

**Background:**

Side population (SP) assay identifies cells with dye/drug extrusion ability, a characteristic of stem cells. Here, we determined if SP cells exist in a verified cell line originating from recurrent nasopharyngeal carcinoma (NPC) and a xenograft established from recurrent metastatic NPC. These cells were evaluated for stem-like properties via functional assays as well as for tumourigenicity.

**Methods:**

We used Hoechst 33342 to identify the SP from non-SP (NSP) cells in HK1 NPC cell line and xeno-284 NPC xenograft. The cells were assayed for *in vitro* characteristics of cancer stem cells (CSC), gene expression and tumourigenicity ability. Student’s t test was used to test for significance.

**Results:**

Five to ten percent and less than 0.5% of HK1 and xeno-284 NPC cells, respectively, were SP cells. Fumitremorgin C (FTC), as opposed to verapamil, was effective in causing the cells to retain Hoechst 33342 dye. HK1 SP cells formed more holoclones, had more aldehyde dehydrogenase (ALDH) activity, divided asymmetrically and contained slow-proliferating cells. *ABCG2*, *SOX2*, *TERT*, *MYC*, Hedgehog, Notch, TGFβ and Wnt signalling pathway genes were significantly upregulated in the SP cells. However, despite these differences *in vitro*, both HK1 SP and NSP cells had an overall similar tumourigenic potential *in vivo*.

**Conclusions:**

HK1 SP cells were ABCG2-specific as confirmed by FTC inhibition and gene expression data. Despite data from *in vitro* and gene expression experiments suggesting stem-like features, there was no significant difference in tumourigenic potential between SP and NSP cells. We conclude that SP assay alone is not sufficient to identify CSCs in HK1 cells. Our work also suggests the presence of a stem-cell like population among NPC cells which do not display increased tumourigenicity.

**Electronic supplementary material:**

The online version of this article (doi:10.1186/s12935-014-0101-0) contains supplementary material, which is available to authorized users.

## Background

Nasopharyngeal carcinoma (NPC) is the most common malignancy arising from the nasopharynx and its causation is closely associated with the Epstein-Barr virus, environmental as well as dietary factors [1]. Majority of NPC cases present in late stages [2]. The late presentation of the disease is due to the hidden location of the tumour, which could present with either no or apparently trivial symptoms which could be dismissed by patients or even medical professionals [3]. In addition, disease recurrence, therapeutic resistance and metastasis remain major clinical problems [4].

The cancer stem cell (CSC) model hypothesizes that there is a hierarchy within the tumour cell population and only a rare subset of cancer cells has the ability to self-renew and to differentiate, leading to the recapitulation of the original tumour [5]. As such, the spread of CSCs is an important component of the process of metastasis and, reactivation of CSC proliferation is believed to be the underlying cause of disease recurrence.

CSCs are found to behave differently from the rest of tumour cells; amongst others, they have enriched tumour-forming potential, and have efficient drug extrusion systems to evade most chemotherapeutic drugs [6]. These cells undergo asymmetric divisions to give rise to daughter cells: one which will be stem-like and, the other which does not show stem cell characteristics. Some CSC-enriched subpopulations were slower in proliferation (reviewed in Moore *et al*. [7]), whilst others reported an equal or rapid proliferation rate than the non-CSC subpopulations [8,9].

As drug extrusion is one of the properties for CSC, several groups had adapted the original side population (SP) assay by Goodell *et al.* [10] for identification of putative stem cells and progenitors in solid tumours [11-13]. The ability of SP cells to extrude the Hoechst 33342 dye, causing them to appear as a dimly stained side population in flow cytometry dot plots, is dependent on the activity of the ATP-binding cassette (ABC) transporter family which includes ABCB1, ABCC1 and ABCG2 [14]. Verapamil is a potent inhibitor for ABCB1 which also weakly inhibits ABCG2 activities, while fumitremorgin C (FTC) specifically inhibits ABCG2 [15,16]. By adding one of these inhibitors into the SP assay, one can determine the type of ABC transporter protein which is responsible for the dye extrusion activity. Prior to this report, there was an earlier publication on the use of SP assay in well- and poorly-differentiated NPC cell lines which indicated that putative CSC in these cell lines may be related to ABCB1 activities [17]. However, *in vivo* tumourigenicity assay was performed for only 4 weeks and the identity of the cell line chosen to perform downstream functional experiments was questioned in a later publication [18].

NPC HK1 is a cell line established from a well-differentiated recurrent NPC sample [19], while xeno-284 is a xenograft line established in our laboratory from a poorly differentiated recurrent metastatic NPC sample. In this study, we first tested for the presence of the SP subpopulation in HK1 and xeno-284 NPC cells, followed by sorting of SP and non-SP (NSP) subpopulations for comparison of *in vitro* clone morphology, cell division and proliferation. Aldehyde dehydrogenase (ALDH) flow-staining was carried out to determine the level of ALDH activity in the sorted cells. Gene expression studies were also performed to identify stem cell related genes and pathways which may be responsible for the observations. Finally, *in vivo* tumourigenicity experiments were performed for duration of up to 7 weeks to evaluate the tumour-initiating ability of SP and NSP cells.

## Results

### HK1 contains SP cell subpopulation

**Figure 1:**
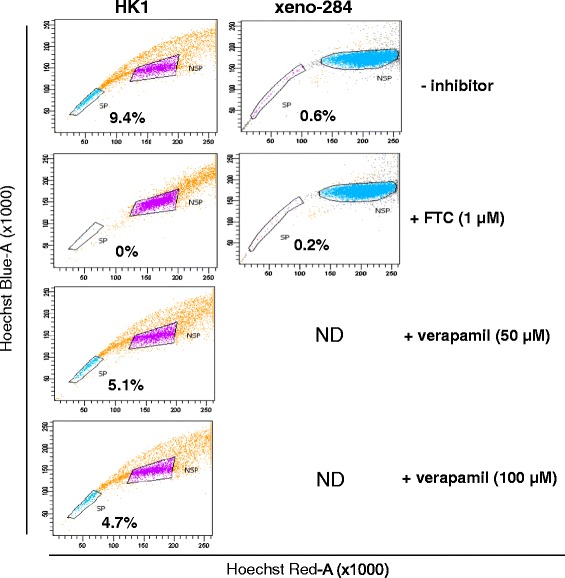
**Identification of side population in NPC cells.** Representative dot plots of HK1 and xeno-284 NPC cells stained with Hoechst 33342 dye, with and without inhibitor. The inhibitory effect of FTC at 1 μM was more evident in HK1 cells as compared to verapamil at both concentrations of 50 and 100 μM. ND: not done.

The identity of the HK1 NPC cells used was validated by short tandem repeat (STR) profiling to be identical to the HK1 cells used by others [18] (Additional file 1). The SP phenotype as identified by low Hoechst 33342 blue/red fluorescence intensity was detected in 5-10% of HK1 cells (Figure 1). The loss of the SP population with addition of FTC but not verapamil, suggested that ABCG2 was the functional ABC transporter in these SP cells (Figure 1). Compared to HK1, xeno-284 cells had very few (less than 0.5%) or no SP cells during replicate runs (Figure 1). As such, only HK1 SP and NSP cells were used for subsequent downstream experiments.

### HK1 SP cells form holoclones during *in vitro* culture

**Figure 2:**
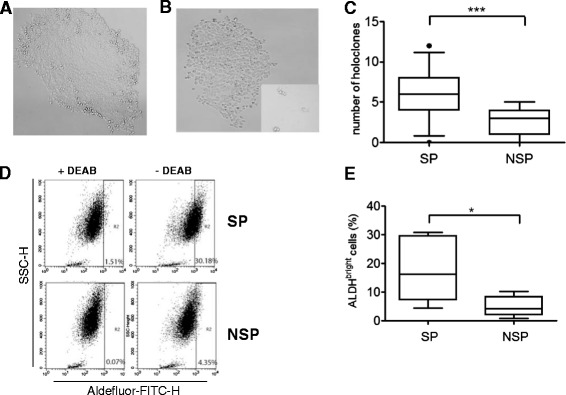
**SP subpopulation enriches for stem-cell like phenotype in** ***in vitro*** **assays. (A)** During *in vitro* culture, majority of SP cells formed holoclones with individual cells clustering tightly and forming a well-defined clone border. **(B)** NSP cells tended to form loose clusters of meroclones or, paraclones (inset) with tiny clusters of cells situated far from each other. All photos were taken on day 9 post-sorting; original magnification 100X. **(C)** SP cells formed more holoclones than NSP cells during *in vitro* culture (p < 0.0001). **(D)** The gating for the ALDH^bright^ population was set by a control tube with the addition of DEAB, an ALDH inhibitor. **(E)** SP cells showed higher presence of ALDH^bright^ cells (18.08 ± 11.46%) than NSP cells (5.10 ± 3.56%) (p < 0.05).

Sorted SP and NSP cells exhibited different growth patterns. After a week of *in vitro* culture in fully-supplemented RPMI medium, most of the SP cells grew into holoclones which formed tightly-clustered cells with well-defined clone borders (Figure 2A). Clones established by the NSP cells primarily consisted of slightly-scattered cells with irregular borders (meroclones) and/or tiny clusters of cells which did not display much growth (paraclones) (Figure 2B). Repeated experiments showed that SP cells formed more holoclones than NSP cells (p < 0.0001; Figure 2C).

### HK1 SP cells express higher aldehyde dehydrogenase activity

The ALDEFLUOR staining kit used in this study detects the enzymatic activity of ALDH1, a marker of stemness [20]. Cells incubated with the ALDEFLUOR substrate (BAAA) and the ALDH inhibitor (DEAB) were used to define the ALDH^bright^ region (representative plots as shown in Figure 2D). The SP cells showed a significantly higher population of ALDH^bright^ cells (18.08 ± 11.46%) than NSP cells (5.10 ± 3.56%) (Figure 2E).

### HK1 SP cells divide asymmetrically into SP and NSP cells during *in vitro* culture

**Figure 3:**
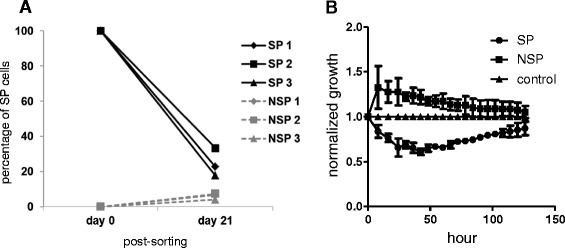
**SP cells grow at a slower rate compared to NSP cells. (A)** Upon relabelling with Hoechst 33342 on day 21 post-sorting, recultured SP cells had divided asymmetrically into SP and NSP phenotypes. Recultured NSP cells had poorer asymmetric division ability. **(B)** Impedance-based cell growth assay indicated that the normalized growth rate for SP cells was lower than NSP cells.

The ability of SP and NSP cells to grow and divide asymmetrically *in vitro* was examined by re-evaluating the percentages of SP and NSP fractions in the sorted cells. After three weeks of culture, the SP sorted cells had divided into both SP and NSP cells, with only 24.57 ± 7.97% of the cells still remaining as SP cells. On the other hand, 6.07 ± 1.74% of SP cells had reappeared in the NSP sorted cells (Figure 3A).

### HK1 SP cells proliferate slower than NSP cells

There was a significant difference in the normalized growth rates between SP and NSP cells in the impedance-based assay (p < 0.0001; Figure 3B). SP cells grew at a slower rate as compared to NSP cells especially at the first 72 hours of observation. At the end of the experiment, growth rates of the SP cells and NSP cells were getting similar to the growth rate of control cells.

### Analysis of stem cell related genes in HK1 SP and NSP cells

**Figure 4:**
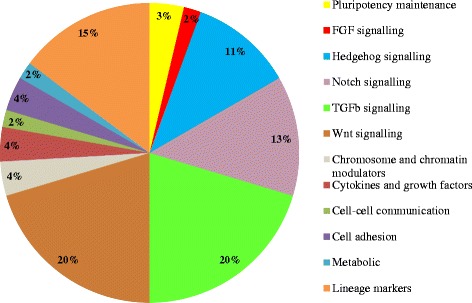
**SP subpopulation enriches for cells expressing stem-cell related genes.** Combined data from Stem Cell PCR Array and Stem Cell Signaling PCR Array were clustered according to gene function or signaling pathways. Gene expression data were normalized to five housekeeping genes via geometric mean calculation. Only genes which were significantly upregulated by at least 2 fold, with raw Ct values of less than 35 are shown here. None of the downregulated genes fulfilled the selection criteria of at least 2 fold change and p < 0.05. Some of the genes appeared in more than 1 gene function or signaling pathway (refer to Additional file 2 for details).

A total of 168 genes related to stem cell identification and/or involved in key stem cell pathways were analysed by quantitative RT-PCR. Fifty genes were significantly upregulated by at least 2 fold in SP cells compared to NSP cells; amongst them were *ABCG2*, *SOX2*, *MYC*, *TERT* and, members of the Hedgehog (*GLI1-3*, *PTCH1, PTCHD2* and *SUFU*), Notch, TGFβ (*ACVR1B*, *SMAD1*, *SMAD7* and *LTBP2-3*) and Wnt (*FZD2*, *FZD6-7*, *BCL9* and *BCL9L*) signalling pathways (Figure 4; Additional file 2). None of the genes tested were significantly downregulated in SP cells.

### HK1 SP subpopulation does not display significant enrichment for tumour-initiating cells in an *in vivo* xenograft assay

As the *in vitro* experiments suggested that the SP subpopulation enriched for cells with stem-like phenotype, we proceeded to determine whether this subpopulation would enrich for tumour-initiating cells in an *in vivo* assay.

**Figure 5:**
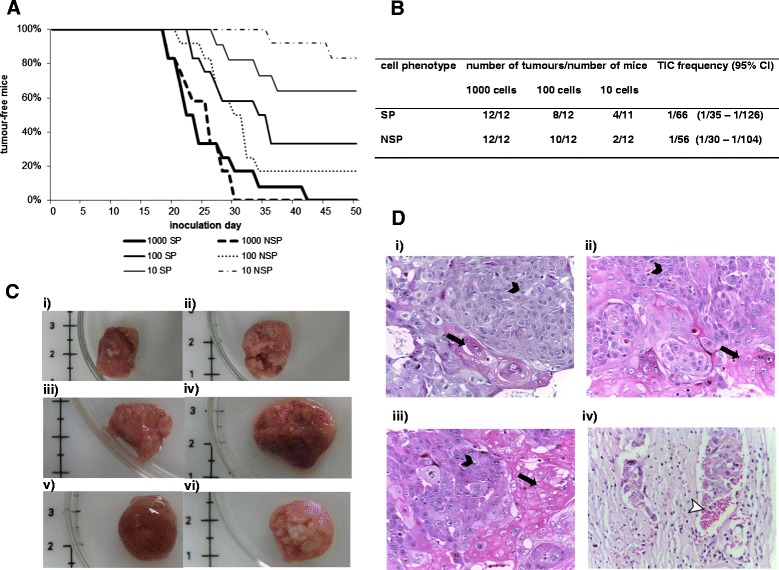
***In vivo*** **tumour growth analysis of SP and non-SP (NSP) cells isolated from HK1 NPC cell line. (A)** SP or NSP cell inoculation in nude mice. Distinct difference in tumour latency was observed in the lowest inoculated cell number (10 cells). **(B)** Limiting dilution analysis of SP and NSP tumours. Tumour-initiating cells (TICs) were similarly enriched in both SP and NSP cells (p > 0.05). **(C)** Representative gross morphology of tumours inoculated with (i) 1000 SP cells, (ii) 1000 NSP cells, (iii) 100 SP cells, (iv) 100 NSP cells, (v) 10 SP cells and (vi) 10 NSP cells. **(D)** H & E photos of tumours after inoculated into nude mice with (i) unsorted HK1 cells, (ii) SP cells and (iii) NSP cells. Tumours derived from SP and NSP cells showed similar histomorphology to unsorted HK1 cells (black arrowhead pointing to squamous carcinoma; long arrow pointing to keratinization). (iv) A SP cell derived tumour showed vascular invasion (white arrowhead pointing to the invasion of SP cells into a blood vessel). All photos at original magnification 400X.

A pilot study showed that 1000 unsorted HK1 cells could form tumours in nude mice (data not shown). To determine the tumour-forming ability of SP and NSP cells, 1000, 100 and 10 cells were inoculated subcutaneously into nude mice (Figure 5A). Three out of four tumours from inoculation of 10 SP cells which were detected earlier than the first tumour from inoculation of NSP cells of the same number suggested that SP cells may have a growth advantage compared to NSP cells. The growth advantage was however lost at higher inoculations of 100 and 1000 cells. Limiting dilution analysis using the Extreme Limiting Dilution Analysis (ELDA) software showed that the estimated number of tumour-initiating cells based on the *in vivo* assay was not significantly different between the SP and NSP subpopulations, implying that the difference in the potential of SP and NSP cells to form tumours was not significant (Figure 5B). Both SP and NSP tumours showed similar histomorphology as seen in unsorted HK1 cells grown in mice (Figure 5C). Histologically, there was also no substantial difference between SP and NSP tumours in the degree of differentiation, stromal reaction and cell pleomorphism (Figure 5D), except for one SP tumour which showed vascular invasion (Figure 5 Div).

## Discussion

CSCs are frequently identified by the presence of surface antigens/markers associated with self-renewal, differentiation, proliferation, metastasis and/or drug resistance characteristics, having increased ALDH activity or through their ability to extrude drugs by ABC transporters. However, these approaches may not be universal in identifying the same phenotype (s) of CSCs within a particular cancer (reviewed in [21]). In NPC, different CSC markers had been reported from the same cell lines generated from primary NPC cases (Additional file 3) [17,22-25]. In this study, we aim to evaluate the presence of CSCs in a cell line established from recurrent NPC by the SP approach.

Results from our various *in vitro*-based assays indicate that the SP cells of HK1 were stem-like. During a week’s growth in fully-supplemented RPMI medium, both SP and NSP cells exhibited different morphology of the colonies in *in vitro* culture. SP cells grew into tightly packed clusters of holoclones, a particular clonal growth morphology closely associated with stem cells and self-renewal ability, as opposed to NSP cells which mostly grew as meroclones and/or paraclones [26-28]. SP cells in HK1 were also found to be significantly enhanced with ALDH activity, which is known to enhance cancer cell survival and therapy resistance [20]. Gene expression analysis showed upregulation of *ABCG2* mRNA level in HK1 SP cells which further indicated the type of ABC transporter being studied here. These SP cells also showed increased mRNA levels for *SOX2*, *MYC* and *TERT* which were associated with CSC biology and maintenance of pluripotency state [29,30]. Over-expression of Hedgehog pathway genes such as *GLI1*, *GLI2*, *GLI3FL, PTCH1* and *PTCHD2* was in line with reports that Hedgehog pathway can induce *ABCG2* expression [31] and it was activated in stem-like cells in EBV positive NPC [23,32].

Nonetheless, there were other findings in our study which seemingly suggested that the stem-like characteristic of SP cells in HK1 may be transient. SP cells of HK1 grew slower in culture which is in line with reports that CSCs from various tumours had slower proliferation rate and hence evaded chemotherapeutic agents which targeted fast growing cells [33,34]. However, the slower rate gradually diminished after a week (Figure 3B). Also, in long term culture, NSP cells gave rise to SP cells (Figure 3A). Together, these two time course *in vitro* experiments (proliferation assay and asymmetry assay) indicated that the difference between SP and NSP sorted cells may be transient, as both type of cells could interconvert [17,35].

The indication that SP cells can only maintain a transient stem-like phenotype in HK1 might well explain the discordance of our *in vitro* results with *in vivo* results. Although there was a growth advantage of SP cells in the lowest inoculation group of mice, an overall comparable tumourigenic potential of both SP and NSP cells was recorded after 7 weeks of *in vivo* transplantation (Figure 5). The discordance of *in vitro* and *in vivo* results is not uncommon, as it has been reported in studies utilizing SP approach to identify CSC in thyroid cancer [36] and cervical cancer [37] as well as studies using marker approach to identify CSC in NPC [25] and colorectal cancer [38]. In our study, although HK1 SP cells were significantly enriched for ALDH^bright^ cells, there were 5% of ALDH^bright^ cells in NSP cells. Wu *et al*. had reported that ALDH positive cells are CSCs in NPC [22]. If ALDH positive cells were also CSCs in recurrent NPC, this would lend credence to our observation of HK1 NSP cells’ ability to generate tumour growth may be partially due to the subset of ALDH positive cells in them.

Our negative findings in *in vivo* study suggested that SP approach in HK1 cells only enriches for a certain subtype with stem-cell features but not bona fide CSCs as depicted by both SP and NSP subpopulations’ ability to confer tumour growth in mice. This is in contrast to an earlier report that showed significant tumour growth difference of SP cells over NSP cells in NPC [17]. The disparity may be related to firstly, the type of NPC cell line being studied. Wang *et al*. utilized CNE-2 cells which had been reported to be contaminated with a partial genome of HeLa, whereas HK1 NPC cells used in this study was verified by STR profiling to be free from HeLa cell contamination [18]. Secondly, we used FTC as a dye efflux inhibitor to identify ABCG2-specific SP cells from HK1 and xeno-284 cells, instead of verapamil, a classic inhibitor for ABCB1-expressing cells [15,16] which showed no inhibition in our study but was used in the above mentioned study [17]. Our negative finding was in line with Zhang *et al*. which reported that ABCG2 was not a CSC marker for NPC [25]. Lastly, the length of tumour assessment time in *in vivo* was different. Mice inoculated with CNE-2 SP and NSP cells were euthanized 4 weeks after inoculation, whereas our mice were observed for 7 weeks. As the incubation time was relatively short, there remains a possibility of CNE-2 NSP cells giving rise to tumour in mice after 4 weeks of inoculation.

## Conclusions

Our study shows that ABCG2-specific SP cell subpopulation is found in HK1 NPC cells, a cell line generated from well-differentiated recurrent NPC but they are minimal in xeno-284 cells, a xenograft derived from poorly differentiated metastatic NPC. In spite of *in vitro* evidence proposing that CSCs from HK1 can be identified by the SP approach, *in vivo* validation study showed that both SP and NSP cells of HK1 had similar tumourigenicity potential. Therefore, we conclude that the SP approach alone cannot identify CSCs accurately in HK1 cells.

## Materials and methods

### Cell line and culture conditions

HK1 NPC cells were cultured in fully-supplemented RPMI 1640 medium containing 10% fetal calf serum and 1% penicillin/streptomycin (Gibco, NY, USA) in a 5% CO_2_ incubator at 37°C. The cells were confirmed to be mycoplasma-free by periodical testing with Venor GeM Mycoplasma Detection Kit for Conventional PCR (Minerva Biolabs, Berlin, Germany).

### Digestion of xenograft sample

Xeno-284 NPC xenograft was freshly harvested and rinsed with cold sterile PBS supplemented with 1X antibiotic/antimycotic (Gibco, NY, USA). After removing visible blood capillaries and/or necrotic tissue, the xenograft tissue was minced finely in the presence of 1X collagenase/dispase solution (Roche, Mannheim, Germany). The mixture was incubated for 60 min in a 5% CO_2_ incubator at 37°C with constant agitation. The cell suspension and undigested xenograft pieces were separated by sieving through 40 μm cap strainer. After a brief centrifugation step, the cell pellet was resuspended in RBC lysis buffer (Qiagen, Hilden, Germany) and, the cells were rinsed with sterile PBS prior to cell count and viability check.

### Hoechst 33342 staining assay

HK1 NPC cells at a logarithmic growth phase were detached by Accutase (Millipore, MA, USA) and washed at least once with PBS prior to Hoechst 33342-staining. A modification of Goodell’s method was used to stain both HK1 and xeno-284 NPC cells [10]. Briefly, the cells were re-suspended at a concentration of 1 × 10^6^ cells per mL in warm HBSS buffer (Gibco, NY, USA) supplemented with 2% fetal calf serum and 10mM HEPES (“HBSS+”). Hoechst 33342 dye (Molecular Probes, OR, USA) was added into the cell suspension at a final concentration of 5 μM in the presence or absence of the ABCG2 inhibitor, fumitremorgin C (FTC) at a final concentration of 1 μM (Sigma, MO, USA) or verapamil (ABCB1 inhibitor) at a final concentration of 50 and 100 μM (Sigma, MO, USA). The cells were incubated for 90 min in a 37°C water bath with intermittent mixing. Excessive Hoechst 33342 dye was removed from the cell suspension by washing with cold HBSS+. The resulting pellet was resuspended to a final concentration of 1 – 2 × 10^6^ cells per mL with cold HBSS+. In order to delineate host mouse cells, xeno-284 cells were further stained with H2Kd-PE antibody (1:10, BD Pharmingen, MA, USA) for 30 min at 4°C. Propidium iodide (PI, 2 μM, BD Pharmingen, MA, USA) was added to stained HK1 NPC cells and, 7-AAD (BD Pharmingen, MA, USA) to Hoechst-stained xeno-284 cells for determination of cell viability. The stained cells were analyzed and sorted in a BD FACSAria II SORP cytometer (BD Biosciences, MA, USA) equipped with a 355 nm-UV laser power of 50 mW. The Hoechst 33342 fluorescence was detected via a 405/20 band-pass filter (Hoechst Blue) and a 670 long pass filter (Hoechst Red). The sorted SP and NSP cells were collected in fully-supplemented RPMI medium. Presence of cells is reported as percentages of single, viable SP or NSP cells. Subsequent experiments were performed using HK1 NPC cells as xeno-284 NPC xenograft had too few SP cells for sorting.

### Clone morphology assay

Sorted cells were plated at a low cell density of 50 cells per well in a 96-well culture plate containing fully-supplemented RPMI medium. Upon seeding, microscopic inspection of each well was performed to ensure there was no cell clumping. Once the cells were adherent, the culture medium was changed every two days. The numbers of holoclones, meroclones and paraclones were counted on day 8 post-sorting. Data were recorded from three independent experiments with 12 replicate wells per SP or NSP cells in each experiment.

### Aldehyde dehydrogenase (ALDH) assay

Sorted cells were left for overnight recovery in the culture conditions as mentioned above. The cells were detached using Accutase and ALDH staining was performed with the ALDEFLUOR staining kit (Stem Cell Technologies, Vancouver, Canada). Briefly, 0.25 × 10^6^ cells were resuspended in ALDEFLUOR assay buffer containing 1.5 μM ALDH activated substrate, BAAA and incubated for 45 mins at 37°C. Half of each sorted sample was treated with 15 μM DEAB, an inhibitor of ALDH. The stained cells were washed, resuspended in cold ALDEFLUOR assay buffer containing 2 μM PI and analyzed in BD FACSCalibur (BD Biosciences, MA, USA). Data were obtained from five independent experiments.

### Asymmetric division assay

Ten thousand sorted cells were recultured for three weeks in fully-supplemented RPMI medium. Hoechst 33342 staining was performed as described above, and the cells were analyzed in a BD FACSAria II SORP cytometer. Data were recorded from three independent experiments.

### Proliferation assay

Sorted cells were left to recover from the sorting process in a 5% CO_2_ incubator at 37°C for approximately two hours. The “recovered” cells were seeded at 3000 cells per 200 μL into each well of the E-plate 16 (Roche, Mannheim, Germany). Cell index values were recorded over a period of 125 hours with an interval of 1 hour for the first day, followed by every 6 hours for the remaining experiment by the xCELLigence System’s Real time Cell Analyzer (RTCA) DP instrument (Roche, Mannheim, Germany). Cell index values represent measurements of electrical impedance of monitored cells which reflect cell growth (number and viability), morphology and adhesion ability. The cell index values of SP and NSP cells at each time point were then normalized to the control cells (HK1 cells which were only stained with PI and sorted from PI negative gate). Data were obtained from three independent experiments.

### Stem cell gene expression study

Quantitative RT-PCR was performed using Human Stem Cell and Stem Cell Signalling PCR Arrays (SABiosciences, MD, USA). Total RNA was isolated using TRIzol (Invitrogen, CA, USA) and cleaned with RNeasy extraction kit (Qiagen, CA, USA). The quality and concentration of the extracted RNA was determined with NanoDrop 8000 spectrophotometer (Thermo Scientific, DE, USA) and only samples with a 260/280 ratio of ~ 1.8 were used for reverse transcription. Total RNA (100 ng) was reverse-transcribed using the accompanying First Strand Synthesis Kit and cDNA was amplified using SYBR green/ROX master mix in the ABI7500 Fast Real-Time thermal cycler (Applied Biosystems, CA, USA). Genes with Ct values of more than 35 were removed from analysis. Ct value of each gene was normalized against the geometric mean Ct value of five housekeeping genes (Ct _geoHK_), with the formula 2^-dCt^ (dCt = Ct _gene of interest_ – Ct _geoHK_). The normalized ×values were used to calculate fold change ratios between SP and NSP. Data were obtained from three independent experiments.

### *In vivo* tumourigenecity assay

Inoculations of SP and NSP cells into nude mice were performed in a limiting dilution manner: 1000, 100 and 10 cells. Sorted cells were mixed with BD Matrigel Basement Membrane Matrix (BD Biosciences, MA, USA) and inoculated subcutaneously into 5 – 6-week-old nude mice (Balb/c nu/nu). Tumour latency data, defined as the period between the inoculation day to the first day of tumour detection, were recorded. Once a palpable growth was detected, tumour volume was recorded every two days. Tumour volume (mm^3^) was calculated from 0.5 × (width × length^2^). All tumours were harvested once the length or width reached 10 mm or on day 50 post-inoculation. Limiting dilution analysis was performed using the Extreme Limiting Dilution Analysis (ELDA) software (http://bioinf.wehi.edu.au/software/elda/). Data for each group were from three independent experiments. All mice experiments were approved by the Animal Care and Use Committee of the Ministry of Health, Malaysia.

### Haematoxylin and eosin (H & E) staining

Four-micrometre formalin-fixed, paraffin-embedded tissue sections from SP and NSP tumours were mounted on plain glass slides and stained with H & E. Histopathological observations were made under a light microscope by a pathologist and all tumours were confirmed to be NPC.

### Statistical analysis

Data are reported as the mean ± standard deviation (SD) or boxplot with whiskers showing the 5 - 95 percentile. All statistical analyses were performed using paired Student’s t test from the GraphPad Prism 5 software (GraphPad Software Inc., CA, USA), except for clone morphology which used unpaired Student’s t test. A p-value of < 0.05 was deemed to be statistically significant.

## Additional files

Additional file 1:
**DNA fingerprinting profile of HK1 cells.**
Additional file 2:
**List of deregulated genes grouped according to gene function or signalling pathways (≥2 fold changes; raw Ct < 35).**
Additional file 3:
**Identification of CSCs in NPC using various approaches.**
