# Supplementary material for: Evaluation of stem-like side population cells in a recurrent nasopharyngeal carcinoma cell line
Source: Cancer Cell Int. 2014 Oct 9;14:101. doi: 10.1186/s12935-014-0101-0 (PMC4195955; doi:10.1186/s12935-014-0101-0)
Supplement: Additional file 2: — List of deregulated genes grouped according to gene function or signalling pathways (≥2 fold changes; raw Ct < 35). [file 12935_2014_101_MOESM2_ESM.docx]

| **Gene** | **Gene description** | **Genbank Accession No.** | **Fold change** | **p value**  (*p<0.05) |
| --- | --- | --- | --- | --- |
| **Pluripotency maintenance** | | | |  |
| *SOX2*  *STAT3* | SRY (sex determining region Y)-box 2  signal transducer and activator of transcription 3 (acute-phase response factor) | NM_003106  NM_003150 | 2.87  2.02 | 0.002*  0.01* |
| **FGF signalling** | | | |  |
| *FGFR3*  *FGF4*  *FGFR1* | fibroblast growth factor receptor 3  fibroblast growth factor 4  fibroblast growth factor receptor 1 | NM_000142  NM_002007  NM_015850 | 20.45  4.26  -1.90 | 0.03*  0.08  0.20 |
| **Hedgehog signalling** | | | |  |
| *SMO*  *GLI2* | Smoothened, frizzled family receptor  GLI family zinc finger 2 | NM_005631  NM_005270 | 49.86  24.02 | 0.08  0.000* |
| *GLI1* | GLI family zinc finger 1 | NM_005269 | 14.12 | 0.002* |
| *PTCHD2* | Patched domain containing 2 | NM_020780 | 9.76 | 0.02* |
| *SUFU* | suppressor of fused homolog  (Drosophila) | NM_016169 | 6.26 | 0.01* |
| *PTCH1*  *DHH* | Patched 1  Desert hedgehog | NM_000264  NM_021044 | 3.52  3.02 | 0.02*  0.05 |
| *GLI3FL* | GLI family zinc finger 3 | NM_000168 | 2.40 | 0.03* |
| **Notch signalling** | | | |  |
| *NOTCH3*  *NOTCH1* | Notch 3  Notch 1 | NM_000435  NM_017617 | 20.42  6.04 | 0.16  0.04* |
| *PSENEN*  *NOTCH4*  *DLL3*  *DTX1* | presenilin enhancer 2 homolog (*C. elegans*)  Notch 4  Delta-like 3 (Drosophila)  Deltex homolog 1 (Drosophila) | NM_172341  NM_004557  NM_016941  NM_004416 | 5.04  3.30  3.11  2.67 | 0.001*  0.25  0.29  0.30 |
| *JAG1* | Jagged 1 | NM_000214 | 2.50 | 0.01* |
| *DTX2* | Deltex homolog 2 (Drosophila) | NM_020892 | 2.27 | 0.03* |
| *NCSTN* | nicastrin | NM_015331 | 2.26 | 0.004* |
| *DLL1*  *PSEN1* | Delta-like 1 (Drosophila)  presenilin 1 | NM_005618  NM_000021 | 2.21  2.00 | 0.001*  0.06 |
| **TGFβ signalling** | | | |  |
| *RGMA* | RGM domain family, member A | NM_020211 | 74.27 | 0.01* |
| *ENG* | endoglin | NM_000118 | 19.35 | 0.01* |
| *LTBP3* | latent transforming growth factor beta binding protein 3 | NM_021070 | 14.78 | 0.002* |
| *E2F5* | E2F transcription factor 5, p130-binding | NM_001951 | 7.85 | 0.04* |
| *LTBP2* | latent transforming growth factor beta binding protein 2 | NM_000428 | 6.09 | 0.01* |
| *ACVR1B* | activin A receptor, type IB | NM_004302 | 4.90 | 0.003* |
| *SMAD1* | SMAD family member 1 | NM_005900 | 3.49 | 0.01* |
| *EP300* | E1A binding protein p300 | NM_001429 | 3.42 | 0.003* |
| *TGFBRAP1* | transforming growth factor, beta receptor associated protein 1 | NM_004257 | 2.82 | 0.02* |
| *BMPR2*  *BMPR1B* | bone morphogenetic protein receptor, type II (serine/threonine kinase)  bone morphogenetic protein receptor,type IB | NM_001204  NM_001203 | 2.76  2.70 | 0.03*  0.08 |
| *SMAD7*  *RBL2* | SMAD family member 7  retinoblastoma-like 2 (p130) | NM_005904  NM_005611 | 2.05  -2.00 | 0.01*  0.16 |
| **Wnt signalling** | | | |  |
| *LRP5* | low density lipoprotein receptor-related protein 5 | NM_002335 | 5.22 | 0.02* |
| *PYGO2* | Pygopus homolog 2 (Drosophila) | NM_138300 | 5.22 | 0.04* |
| *BCL9* | B-cell CLL/lymphoma 9 | NM_004326 | 4.80 | 0.01* |
| *FZD7*  *TCF7* | Frizzled family receptor 7  transcription factor 7 (T-cell specific, HMG-box) | NM_003507  NM_003202 | 4.48  4.00 | 0.01*  0.10 |
| *FZD2* | Frizzled family receptor 2 | NM_001466 | 3.88 | 0.04* |
| *AXIN1* | Axin 1 | NM_003502 | 3.39 | 0.002* |
| *MYC* | v-myc myelocytomatosis viral oncogene homolog (avian) | NM_002467 | 3.01 | 0.02* |
| *NFAT5* | nuclear factor of activated T-cells 5, tonicity-responsive | NM_006599 | 2.61 | 0.01* |
| *FZD6* | Frizzled family receptor 6 | NM_003506 | 2.41 | 0.004* |
| *PPARD*  *LRP6* | peroxisome proliferator-activated receptor delta  low density lipoprotein receptor-related protein 6 | NM_006238  NM_002336 | 2.09  2.04 | 0.01*  0.10 |
| *BCL9L*  *LEF1* | B-cell CLL/lymphoma 9-like  lymphoid enhancer-binding factor 1 | NM_182557  NM_016269 | 2.02  -2.20 | 0.01*  0.40 |
| **Miscellaneous** | | | |  |
| *NUMB*  *CD4* | Numb homolog (Drosophila)  CD4 molecule | NM_003744  NM_000616 | 42.73  10.54 | 0.35  0.03* |
| *ALPI*  *MYOD1*  *NEUROG2*  *GJB1*  *ACAN* | alkaline phosphatase, intestinal  myogenic differentiation 1  neurogenin 2  gap junction protein, beta 1, 32kDa  aggrecan | NM_001631  NM_002478  NM_024019  NM_000166  NM_001135 | 9.48  7.27  6.05  5.88  5.81 | 0.03*  0.08  0.05  0.06  0.05 |
| *TERT* | telomerase reverse transcriptase | NM_198253 | 5.44 | 0.003* |
| *KRT15* | keratin 15 | NM_002275 | 4.69 | 0.01* |
| *S100B* | S100 calcium binding protein B | NM_006272 | 3.98 | 0.04* |
| *BGLAP*  *CD3D*  *GDF3*  *GDF2*  *CD8A* | bone gamma-carboxyglutamate (gla) protein  CD3d molecule, delta (CD3-TCR  complex)  growth differentiation factor 3  growth differentiation factor 2  CD8a molecule | NM_199173  NM_000732  NM_020634  NM_016204  NM_001768 | 3.81  3.70  3.64  3.12  2.96 | 0.01*  0.11  0.16  0.06  0.47 |
| *T* | T, brachyury homolog (mouse) | NM_003181 | 2.88 | 0.02* |
| *ABCG2*  *GJB2* | ATP-binding cassette, sub-family G (WHITE), member 2  gap junction protein, beta 2, 26kDa | NM_004827  NM_004004 | 2.63  2.63 | 0.003*  0.09 |
| *KAT2A* | K(lysine) acetyltransferase 2A | NM_021078 | 2.62 | 0.002* |
| *JAG1* | Jagged 1 | NM_000214 | 2.50 | 0.006* |
| *BMP1*  *SOX1*  *BMP2* | bone morphogenetic protein 1  SRY (sex determining region Y)-box 1  bone morphogenetic protein 2 | NM_006129  NM_005986  NM_001200 | 2.44  2.23  2.14 | 0.03*  0.27  0.14 |
| *COL1A1* | collagen, type I, alpha 1 | NM_000088 | 2.09 | 0.03* |
| *TUBB3*  *PDX1*  *MME* | tubulin, beta 3  pancreatic and duodenal homeobox 1  membrane metallo-endopeptidase | NM_006086  NM_000209  NM_000902 | 2.04  2.01  -2.68 | 0.03*  0.29  0.12 |
