# Supplementary material for: Evaluation of stem-like side population cells in a recurrent nasopharyngeal carcinoma cell line
Source: Cancer Cell Int. 2014 Oct 9;14:101. doi: 10.1186/s12935-014-0101-0 (PMC4195955; doi:10.1186/s12935-014-0101-0)
Supplement: Additional file 3: — Identification of CSCs in NPC using various approaches. [file 12935_2014_101_MOESM3_ESM.docx]

**NPC cell lines established from Functional assays CSC markers**

**primary NPC specimens SP ALDH CD44 CD133 ABCG2**

CNE-2 ref. 17 ref. 22 ref. 24

5-8F ref. 22 ref. 25*

C666-1 ref. 23

*negative findings
